# Longitudinal Association between *n*-3 Long-Chain Polyunsaturated Fatty Acid Intake and Depressive Symptoms: A Population-Based Cohort Study in Japan

**DOI:** 10.3390/nu10111655

**Published:** 2018-11-03

**Authors:** Chika Horikawa, Rei Otsuka, Yuki Kato, Yukiko Nishita, Chikako Tange, Tomohiro Rogi, Hiroshi Kawashima, Hiroshi Shibata, Fujiko Ando, Hiroshi Shimokata

**Affiliations:** 1 Section of the NILS-LSA, National Center for Geriatrics and Gerontology, 7-430 Morioka-cho, Obu-City, Aichi 474-8511, Japan; otsuka@ncgg.go.jp (R.O.); kyuki@asu.aasa.ac.jp (Y.K.); nishita@ncgg.go.jp (Y.N.); tange@ncgg.go.jp (C.T.); fujikoa@ncgg.go.jp (F.A.); hiroshi@ncgg.go.jp (H.S.); 2 Institute for Health Care Science, Suntory Wellness Limited, 8-1-1 Seikadai, Seika-cho, Soraku-gun, Kyoto 619-0284, Japan; Tomohiro_Rogi@suntory.co.jp (T.R.); Hiroshi_Kawashima@suntory.co.jp (H.K.); Hiroshi_Shibata@suntory.co.jp (H.S.); 3 Faculty of Health and Medical Sciences, Aichi Shukutoku University, 2-9 Katahira, Nagakute-city, Aichi 480-1197, Japan; 4 Graduate School of Nutritional Sciences, Nagoya University of Arts and Sciences, 57 Takenoyama, Iwasaki-cho, Nisshin-city, Aichi 470-0196, Japan

**Keywords:** *n*-3 LCPUFA, depressive symptoms, community-dwelling, cohort, Japanese, NILS-LSA

## Abstract

It remains unclear whether *n*-3 long-chain polyunsaturated fatty acids (LCPUFA) have a preventive effect on depression in the general population. This study investigated the longitudinal association between *n*-3 LCPUFA intake and depressive symptoms in community-dwelling Japanese participants. The participants were aged 40–79 years at baseline in the cohort study, wherein examinations, including the assessment of depressive symptoms and nutritional status, were biennially conducted from 1997 to 2012. The subjects (*n* = 2335) who had a Center for Epidemiologic Studies Depression Scale (CES-D) score < 16 at the first examination and who participated in the follow-up study at least once were included in the analysis. The follow-up end point was the first onset (CES-D ≥ 16) or the last examination participation. Hazard ratios (95% CIs) for CES-D ≥ 16 were estimated using the adjusted Cox proportional hazards model. Overall, 22.1% participants showed depressive symptoms during follow-up (average; 8.1 years). Compared with the lowest tertile, the highest HR for EPA was 0.74 (0.60–0.93), and highest and middle HRs for DHA were 0.79 (0.63–0.98) and 0.80 (0.65–0.99) (*P* for trend = 0.009 and 0.032), respectively. Among populations with high fish consumption, higher *n*-3 LCPUFA intake may be associated with a low risk of depressive symptoms.

## 1. Introduction

Depression is a serious public health problem worldwide. The World Health Organization estimates that approximately 322 million people worldwide suffer from depression [1]. The illness is predicted to become one of the top three causes of disability-adjusted life years lost by 2030 [2].

In the approximately 20 years since 1996, the incidence of mood disorders, including depression and bipolar affective disorder, has doubled in Japan, and the number continues to rise [3]. In Europe and the United States, young people are at a higher risk of developing depression than old people [4]. However, recent Japanese surveys have reported that depression occurs frequently among middle-aged and elderly people [5]. People in these age groups may have suffer from functional impairments that occur with age in addition to depression. Dementia is particularly worrisome among such people. Depressive symptoms often present in people with multiple chronic conditions and age-related cognitive impairment [6], as well as in people with early dementia with prodromal signs of cognitive decline [7,8]. Depression has attracted attention as a potential risk factor for dementia. The impact of depression and methods of preventing or treating depression need to be addressed in developed countries with aging societies.

Diet is a well-known factor associated with depression [9,10,11]. This has been particularly reported in studies associated with fish intake [12,13]. Ecologic studies have shown that people living in countries with high fish consumption, such as Japan, have lower rates of age-standardized disability-adjusted life years for depressive disorders [14]. Eicosapentaenoic acid (EPA) and docosahexaenoic acid (DHA) are the two major *n*-3 long-chain polyunsaturated fatty acids (*n*-3 LCPUFA) derived from fish and shellfish; they are essential for maintaining cellular membrane function and affect neurotransmitter release and modify receptor binding [15]. We should pay attention to the potential mechanisms that originate from models for depression with limitation when it comes to the comparability with human depression/depressive symptoms [16,17]; however, experimental studies have shown that these fatty acids have anti-inflammatory [18], antioxidative [19], neuroprotective [20], and neurogenesis effects [21]. These factors may also be important in protecting against depression and inflammation-related diseases [22]. Therefore, we hypothesized that EPA and DHA may be effective against the pathophysiology of depressive symptoms.

According to a recent meta-analysis, the intake of *n*-3 LCPUFA showed favorable effects in patients with major depressive disorder (MDD) [23,24]. Conversely, another review indicated the presence of insufficient evidence for the efficacy of *n*-3 LCPUFA as a treatment for MDD [25]. Thus, findings regarding the effect of *n*-3 LCPUFA intake in patients with MDD are not consistent. Certainly, it is important to be able to treat MDD; however, it is even more important to understand daily nutrition for preventing the risk of depression as the need for prevention has recently increased to become higher than the need for therapeutic management of disease. The meta-analysis of observational studies showed a significant association between *n*-3 LCPUFA and the risk of depression [26]. However, most studies on populations from countries with low fish intake did not indicate a significant linear dose-response association between *n*-3 LCPUFA intake and the risk of depression. Recently, one Japanese cohort study reported that a high intake of fish or *n*-3 LCPUFA protected a Japanese population from MDD [27]. However, these results showed a reverse J-shaped curve as opposed to a simple linear association. Thus, it remains unclear whether the risk of depressive symptoms decreases with higher intake of *n*-3 LCPUFA in the Japanese general population.

In this study, we aimed to investigate the longitudinal association between *n*-3 LCPUFA intake and depressive symptoms in community-dwelling Japanese subjects whose habitual intake of *n*-3 LCPUFA was higher than that in other countries. We particularly focused on middle-aged and elderly people who are at higher risk for depression. The intake of fatty acids other than *n*-3 LCPUFA was also examined because it was necessary to ascertain whether findings regarding any association were specific to *n*-3 LCPUFA.

## 2. Materials and Methods 

### 2.1. Study Design

The data for the present study was obtained from the National Institute for Longevity Sciences-Longitudinal Study of Aging (NILS-LSA). The NILS-LSA is a Japanese population-based prospective cohort study of normal aging and age-related diseases. The normal aging process was assessed using detailed questionnaires and medical checkups, anthropometric measurements, physical fitness tests and nutritional assessments. The participants in the NILS-LSA of the first wave included approximately 2300 males and females aged 40–79 years [28]. The first wave of the examination began in November 1997 and ended in April 2000. Following the first wave of the examination, participants were followed up every 2 years. The seventh wave of the examination was completed in July 2012, and a postal survey for follow-up is ongoing. Participants in the NILS-LSA included randomly selected, age- and sex-stratified individuals from the noninstitutionalized residents in the National Center for Geriatrics and Gerontology neighborhood areas of Obu City and Higashiura Town in Aichi Prefecture. When participants could not attend the follow-up investigations (because of moving away, death, or other personal reasons), the same number of age- and sex-matched random samples were recruited, excluding individuals aged >79 years. Male and female participants aged 40 years were also newly recruited from the same residential area. Details of the NILS-LSA study have been reported elsewhere [28]. This research is not a clinical trial and is therefore not registered.

### 2.2. Study Population 

**Figure 1:**
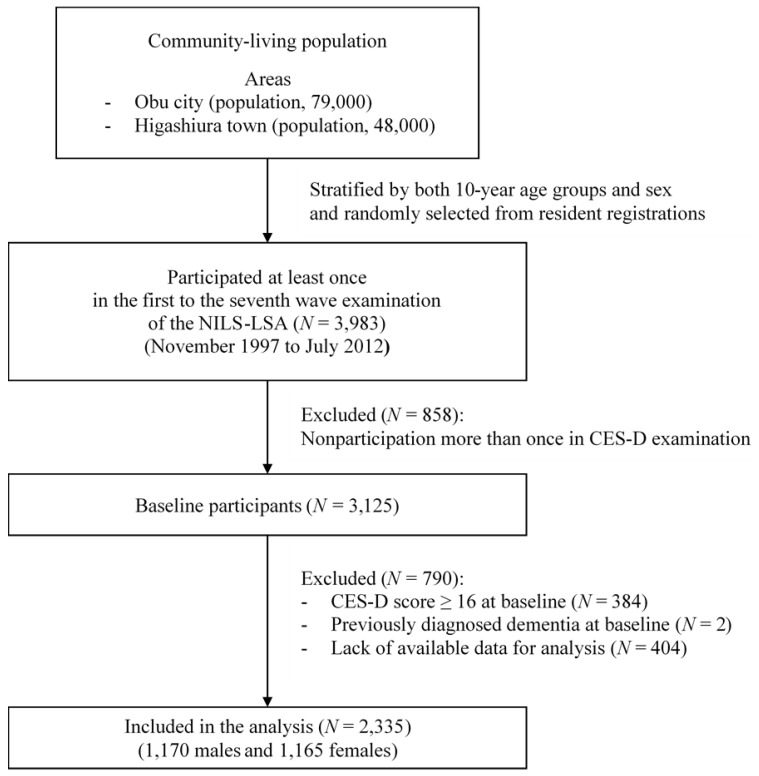
Subjects included in this study. CES-D, Center for Epidemiologic Studies Depression Scale; N, number; NILS-LSA, National Institute for Longevity Sciences–Longitudinal Study of Aging.

The NILS-LSA included 3983 males and females aged 40 years or older who participated in at least one examination from the first to the seventh wave of examinations (from 1997 to 2012). The baseline for each individual was at the first assessment time point by the Center for Epidemiologic Studies Depression Scale (CES-D). The follow-up time was the period from each baseline (the time of each individual’s first CES-D assessment) to the first onset of depressive symptoms or the censored date (the last examination day). We excluded the following individuals: Those who failed to participate more than once in the CES-D assessment; those with CES-D scores ≥ 16 at each baseline; those with a self-reported history of previously diagnosed dementia at each baseline and those with missing data at each baseline for adjusted variables in the statistical analysis. Lastly, on the basis of the exclusion criteria, 2335 subjects (1170 males and 1165 females) were included in the analysis (Figure 1). The study was approved by the Committee for Ethics of Human Research of the National Center for Geriatrics and Gerontology (No. 899-2) and was conducted in accordance with the Declaration of Helsinki. Written informed consent was obtained from all subjects.

### 2.3. Depressive Symptoms

Depressive symptoms were assessed using the Japanese version [29] questionnaire of the CES-D [30]. The participants completed the self-completed questionnaire CES-D at home for assessing their depressed state in the previous week and brought it with them on the examination day. The CES-D questionnaire comprises 20 questions in the following four subscales: Somatic and retarded activity, depressed affect, positive affect and interpersonal relations. Scores range from 0 to 60, with lower scores indicating fewer depressive symptoms. This scale is reportedly a valid and reliable measure of depressive symptoms in the elderly [31]. A cut-off score of ≥16 is used for identifying subjects with relevant depressive symptoms [30]. We defined a CES-D score ≥16 as representative of depressive symptoms. We regarded the first time point at which the CES-D score was ≥16 during the follow-up period as the onset of depressive symptoms.

### 2.4. Nutritional Assessment

Nutritional intakes, such as mean energy intake per day (kcal/day) and fatty acid intake per day (g or mg/day), were assessed using a 3-day dietary record after participation in all waves. The dietary record was completed over three continuous days (two weekdays and one weekend day) [32]. Most subjects completed it at home and returned the records within 1 month. Either the food was separately weighed on a 1-kg kitchen scale (Sekisui Jushi, Tokyo, Japan) before being cooked, or portion sizes were estimated. The subjects used a disposable camera with 27 shots (167 Fuji Film, Tokyo, Japan) for taking photographs of their meals before and after eating. Dieticians used these photographs to complete missing data, and they then telephoned the subjects to resolve any discrepancies or to obtain further information when necessary. Average 3-day food and nutrient intakes were calculated according to the Standard Tables of Foods Composition in Japan 2010 [33]. Alcohol intake in the previous year was assessed using a food frequency questionnaire; trained dieticians interviewed the subjects using this questionnaire.

### 2.5. Other Measurements

A self-completed questionnaire, provided approximately 2 weeks prior to the examination day, was used for collecting information on the history of hypertension, hyperlipidemia, ischemic heart disease, stroke and diabetes (yes/no); education (≤9, 10–12, or ≥13 years of school); employment status (no occupation or household labor/nonregular employment/regular employment); marital status (yes/no) and smoking status (yes/no). The questionnaires were collected on the examination day. Both weight and height were measured on the examination day, and BMI was calculated as the weight in kilograms divided by the square of the height in meters (kg/m^2^). Trained interviewers used a questionnaire for assessing physical activity using questions regarding the intensity and frequency of activity over the preceding year [34]. The mean amount of leisure-time physical activity per day was calculated (metabolic equivalents [MET], MET × min/day).

### 2.6. Statistical Analysis

All statistical analyses were conducted using Statistical Analysis System version 9.3 (SAS Institute, Cary, NC, USA). The confounding variables were sex, age, BMI, educational level, marital status, smoking status, alcohol consumption, energy intake, physical activity, employment status, CES-D score and history of hypertension, hyperlipidemia, ischemic heart disease, stroke, and diabetes at baseline. Differences in characteristics and fatty acid intake between subjects with and without new onset of depressive symptoms were assessed using the χ^2^-test for categorical variables or Student’s *t*-test for continuous variables. To analyze the association between fatty acid intake and risk of depressive symptoms, we performed Cox proportional hazards regression and estimated the hazard ratios (HRs) and 95% CIs of depressive symptoms for tertiles of fatty acid intake using the lowest tertile category as the reference. Trend associations were assessed by entering dummy variables assigned to the tertile of fatty acid intake. In model I, we adjusted for age, sex and energy intake. Model II was further adjusted for the confounding variables, including BMI, educational level, marital status, smoking status, alcohol consumption, physical activity, employment status, CES-D score and previous medical history at baseline. Two-sided *p* values < 0.05 were regarded as indicating statistical significance. 

## 3. Results

### 3.1. Characteristics of the Study Population

The subjects for analysis consisted of 1170 males (50.1%) and 1165 females (49.9%). The mean age at baseline was 56.1 (SD 11.9) years, and 515 subjects (22.1%) were identified as having depressive symptoms (CES-D score ≥ 16) during the follow-up period. The average follow-up time was 8.1 (SD 3.8) years. The characteristics of subjects with and without the onset of depressive symptoms are shown in Table 1. Compared with subjects who presented without new onset of depressive symptoms, subjects who presented with new onset of depressive symptoms were significantly more likely to be female, had fewer years of education, and had a higher CES-D score at baseline. Marginally significant differences were observed for marital status and history of diabetes.

### 3.2. Differences between Fatty Acid Intake with and without the Onset of Depressive Symptoms 

Table 2 shows the fatty acid intake level at baseline according to the presence or absence of new onset of depressive symptoms during the follow-up period. The fatty acid intake level at baseline, including EPA (*p* = 0.049), DHA (*p* = 0.020), and *n*-3 PUFA (*p* = 0.040), was significantly different in subjects with and without onset of depressive symptoms. Subjects with the onset of depressive symptoms had significantly lower fatty acid intake than those without depressive symptoms. However, the intake of α-linolenic acid (ALA) among *n*-3 PUFAs and *n*-6 PUFA did not significantly differ between groups.

### 3.3. Risk of Depressive Symptoms with Increased Intake of Fatty Acids

Table 3 and Table 4 present the HRs and 95% CIs of depressive symptoms for tertiles of fatty acid intake, using the low tertile category as the reference. The risk of depressive symptoms was inversely associated with the increased intake of *n*-3 PUFAs, with a significant association in the high tertile compared with the low tertile (Table 3). No associations were noted between intake levels of any fatty acid other than *n*-3 PUFAs and the onset of depressive symptoms. No significant associations were observed with *n*-6 PUFA levels, but the *n*-6:*n*-3 ratio showed a significant trend of association with the onset of depressive symptoms.

Table 4 shows the risk of depressive symptoms according to the tertile of individual fatty acid intake. In the crude model, the increased intake of EPA and DHA showed an inverse association with the onset of depressive symptoms (*p* for trend = 0.016 and 0.023, respectively). The crude HRs for EPA and DHA in the highest tertile were significantly lower than those for EPA and DHA in the lowest tertile (EPA: HR 0.77, 95% CI 0.62–0.95; DHA: HR 0.79, 95% CI 0.64–0.97). The HR for DHA was also significant in the middle tertile compared with the lowest tertile. In model I, which was adjusted for sex, age, and energy intake, the result was similar to that in the crude model. Furthermore, these significant associations were maintained even in model II after adjusting for more covariates, including baseline CES-D score, BMI, educational level, marital status, smoking status, alcohol consumption, physical activity, employment status, and history of hypertension, hyperlipidemia, ischemic heart disease, stroke, and diabetes. After adjustment for all covariates, EPA was associated with a lower HR for onset of depressive symptoms (high tertile vs. low tertile: HR 0.74, 95% CI 0.60–0.93). Similarly, higher DHA was associated with a lower HR for onset of depressive symptoms (high tertile vs. low tertile: HR 0.79, 95% CI 0.63–0.98; middle tertile vs. low tertile: HR 0.80, 95% CI 0.65–0.99). Moreover, the HR showed a decreasing trend with level of intake of both EPA (*p* for trend = 0.009) and DHA (*p* for trend = 0.032). There were no significant associations for other fatty acids.

## 4. Discussion

In the present study, higher intakes of EPA and DHA were shown to be effective in reducing the risk of depressive symptoms in Japanese middle-aged and elderly community-dwelling subjects. To the best of our knowledge, this is the first study demonstrating a decreased risk in response to the intake levels of EPA and DHA in a population with a high fish intake. This association was specific to EPA and DHA. 

During 8.1 years of follow-up, 22.1% of participants had an onset of depressive symptoms. This rate was higher than that in our previous cross-sectional study (12.5%) and in other reports regarding Japanese middle-aged and elderly subjects [35]. The difference between cross-sectional and longitudinal studies may partially contribute to this discrepancy.

In a cohort study that used CES-D for assessing the depressed state and observed subjects for a similar follow-up period, the incidence of depressive symptoms ranged from 11.3% to 29.2% [6,36,37].

Japanese people consume a large amount of fish and shellfish, which are the main sources of EPA and DHA [38,39], and the subjects in the present study had a high intake of these fatty acids. The median values for EPA and DHA consumption were 243 and 469 mg/day, respectively. The intake of EPA and DHA in Japan are approximately 10 times higher than those in the United States and Australia, where the fish intake is lower [38,40]. Other Japanese studies of middle-aged and elderly subjects also found high intake levels of *n*-3 LCPUFA (range, 0.8–1.0 g/day) [27,41,42]. In cohort studies similar to the present study, high intakes of *n*-3 LCPUFA were found in Finland (0.5 g/day) and Spain (0.9 g/day) [43,44], whereas most cohort studies in other Western countries, such as the United States and Australia, have found an intake of approximately 0.1 g/day [36,40,45].

Ecological studies have shown that age-standardized disability-adjusted life years among subjects with depressive disorders are lower in countries in which people consume more fish [12]. However, in cohort studies in countries with higher fish intake, no clear association between *n*-3 LCPUFA intake levels and depressive symptoms has been observed. We initially considered that the lack of clear evidence for this association may be associated with the saturation effect of *n*-3 LCPUFA levels on depressive symptoms, but a dose-related association between *n*-3 LCPUFA levels and depressive symptoms was demonstrated in a cross-sectional study using serum samples from Japanese subjects [35]. EPA and DHA intake levels in regions with low fish intake may be insufficient to impact depressive symptoms.

Our research evaluated EPA and DHA intake levels using a 3-day dietary record, whereas numerous Western cohort studies use Food Frequency Questionnaires (FFQs) [6,44,46] instead of dietary records. Another Japanese cohort study has also used the FFQ [27]. The dietary intake method used in our study reportedly provides more accurate intake levels than the FFQ [47,48]. Differences in accuracy between dietary evaluation methods may influence the results of different studies.

Variations in subject characteristics may also explain discrepancies between study findings. The participants in the present study was from a general, randomly selected, community-dwelling population, whereas most previous reports included participants from cohort-based intervention studies. Intervention studies may be associated with selection bias because of the precise selection criteria. Among the cohort studies of the ingestion of EPA and DHA and the risk of depressive symptoms, only two studies in Spain [44] and Japan [27] were conducted in areas with high fish intake. The findings of these studies were found to be partially significant and not dose-dependent. These results may be because of the insufficient research period. The follow-up period of the Spain cohort study was approximately 2 years, which may be too short a period to observe the occurrence of an event. Conversely, the follow-up period in the Japanese study was 25 years, which may be too long. In addition, these studies either used specific educational levels for study inclusion [44] or did not consider education as a confounding variable [27]. The educational level is associated with the incidence of depressive symptoms [49,50]. Thus, selection bias is more likely to have occurred in these studies. The Spain study also included onset of symptoms of anxiety besides depression, and the rate was 67% in all subjects; thus, the relevance of depressive symptoms may not have been considered. Depression may also be a critical complication of stroke and diabetes [51,52,53]; however, few studies have adjusted for these variables. Future studies should aim to identify all factors associated with depression and the intake of fatty acids.

There were few differences between EPA and DHA in the present study, as the tertiles of both fatty acid concentrations showed an association with a lower HR for depressive symptoms, with significant trends. A recent meta-analysis provided evidence that EPA may be more efficacious than DHA in treating depression [54]. Another meta-analysis suggested that only regimens containing over 60% EPA had a highly significant effect in the treatment of depression [55]. DHA is also a major component of brain neurons [56] and plays an important role in maintaining regular brain function [57]. However, an intervention study indicated the possibility of a better effect of DHA than EPA on depression [58]. In our study, no significant associations were observed with *n*-6 PUFA levels, but the *n*-6:*n*-3 ratio showed a significant trend of association with the onset of depressive symptoms as is the case with numerous other reports. A high dietary *n*-6:*n*-3 ratio associated with increased risk of depression has been often reported [59]. But most reports only reflect the results derived from the inverse associations of *n*-3 LCPUFA with the risk of depression as in our study. Another article concluded that increased intake of oleic acid was associated with reduced risk of severe depressed mood while increased intake of linoleic acid was associated with increased risk [60]. The results of the present study were inconsistent with those of this study because we observed no association with oleic acid and linoleic acid. Higher *n*-3 LCPUFA intake of Japanese than people of other countries may affect these differences, but the details remain unclear.

This study has several limitations. First, we did not have a diagnosis of clinical depression. Although the CES-D is a valuable assessment scale for studying the association between depressive symptoms and several variables, it is not a clinical diagnostic tool [30]. Second, our nutritional assessment method is more accurate than other methods [47,48], but it still may not be sufficient in this research. Because daily fluctuations in EPA and DHA intake within each subject were large, a 3-day observation period for dietary intake may have been insufficient. However, the fluctuation may have had little influence on the results because the estimated intake was based on the cohort population and not on individuals. Third, there was no control for time-varying covariates. We adjusted only for covariates obtained at the baseline. This may induce systematic bias that yields an underestimate of HR. Fourth, we did not control for multiple comparisons because we particularly focused on *n*-3 LCPUFA on the basis of our hypothesis. But fatty acids other than *n*-3 LCPUFA were also examined because it was necessary to ascertain whether findings regarding any association were specific to *n*-3 LCPUFA. Fifth, the small effect size was not large enough to make definite conclusions. However, systematic review and meta-analysis of results obtained from observational studies support the hypothesis that dietary fish-derived *n*-3 PUFA intake is associated with a decreased risk of depression [26]. Although the effect size was small, the habitual high fish-derived fatty acid intake may be clinically effective in preventing depressive symptoms.

## 5. Conclusions

To conclude, the present study suggests that EPA and DHA intake may have a favorable effect on depressive symptoms in Japanese subjects with higher intake levels of *n*-3 LCPUFA. Lower intake levels may lead to more depressive symptoms in subjects in countries with lower *n*-3 LCPUFA intake. Daily intake of these fatty acids may be beneficial. Further longitudinal and intervention studies are needed to elucidate any preventive effects of *n*-3 LCPUFA against depressive symptoms.

## Figures and Tables

**Table 1:** Baseline characteristics of subjects according to the onset of depressive symptoms in the follow-up study (numbers and percentages, mean values and standard deviations).

Characteristics	Total		CES-D ≥ 16		CES-D < 16		*p*-Value *
			Subjects with New Onset of Depressive Symptoms		Subjects without New Onset of Depressive Symptoms		
	Mean	SD	Mean	SD	Mean	SD	
No. of subjects (*n*)	2335		515		1820		
Sex (male/female) (*n*)	1170/1165 (50.1/49.9%)		227/288 (44.1/55.9%)		943/877 (51.8/48.2%)		0.002
Age (years)	56.1	11.9	56.4	11.6	56.1	12.0	0.62
BMI (kg/m^2^)	22.9	3.0	22.8	3.2	23.0	3.0	0.44
CES-D	5.4	4.2	8.0	4.2	4.7	3.9	< 0.001
Educational level (*n*)							
≤9 years	581 (24.9%)		150 (29.1%)		431 (23.7%)		
10–12 years	950 (40.7%)		204 (39.6%)		746 (41.0%)		0.032
≥13 years	804 (34.4%)		161 (31.3%)		643 (35.3%)		
Unmarried (*n*)	281 (13.7%)		74 (14.4%)		207 (11.4%)		0.07
Current smoker (*n*)	481 (20.6%)		108 (21.0%)		373 (20.5%)		0.81
Energy intake (kcal/day)	2136	428	2123	408	2140	433	0.44
Alcohol consumption † (mL/day)	9.7	16.1	8.9	16.5	10.0	16.0	0.18
Total physical activity	2.0	0.2	2.0	0.2	2.0	0.2	0.87
(Mets * min/1000/day)							
Employment (*n*)							
Unemployed or household labor	877 (37.6%)		198 (38.5%)		679 (37.3%)		
Nonregular employment	407 (17.4%)		102 (19.8%)		305 (16.8%)		0.15
Regular employment	1051 (45.0%)		215 (41.8%)		836 (45.9%)		
Medical history ‡ (*n*)							
Stroke	47 (2.0%)		12 (2.3%)		35 (1.9%)		0.56
Hypertension	511 (21.9%)		123 (23.9%)		388 (21.3%)		0.21
Ischemic heart disease	190 (8.1%)		49 (9.5%)		141 (7.8%)		0.20
Hyperlipidemia	357 (15.3%)		83 (16.1%)		274 (15.1%)		0.56
Diabetes mellitus	155 (6.6%)		25 (4.9%)		130 (7.1%)		0.07

*n*, number; CES-D, Center for Epidemiologic Studies Depression Scale. * Continuous variables, Student’s *t*-test; categorical variables, χ^2^ test. † Alcohol consumption was converted into ethanol content. ‡ Past and present illness.

**Table 2:** Baseline intake and ratios of fatty acids according to the onset of depressive symptoms in the follow-up study (mean values and standard deviations).

Fatty Acid		Total		CES-D ≥ 16		CES-D < 16		*p*-Value *
				Subjects with New Onset of Depressive Symptoms		Subjects without New Onset of Depressive Symptoms		
		Mean	SD	Mean	SD	Mean	SD	
SFA	(g/day)	16.6	5.7	16.4	5.3	16.6	5.8	0.50
MUFA	(g/day)	20.7	7.0	20.6	6.6	20.7	7.1	0.67
PUFA	(g/day)	13.4	4.0	13.2	3.9	13.4	4.0	0.46
*n*-3 PUFA	(g/day)	2.5	1.0	2.4	0.9	2.5	1.0	0.040
DHA	(mg/day)	552.2	377.9	519.4	356.2	561.5	383.4	0.020
EPA	(mg/day)	301.0	240.2	282.6	229.0	306.2	243.1	0.049
ALA	(mg/day)	1438	532	1425	527.2	1442	533.4	0.53
*n*-6 PUFA	(g/day)	10.8	3.4	10.8	3.3	10.8	3.4	0.79
ARA	(mg/day)	176.8	65.5	174.7	61.6	177.5	66.6	0.38
LA	(g/day)	10.6	3.4	10.5	3.3	10.6	3.4	0.93
*n*-6:*n*-3		4.7	1.6	4.8	1.5	4.7	4.6	0.048

ALA, α-linolenic acid; ARA, arachidonic acid; CES-D, Center for Epidemiologic Studies Depression Scale; DHA, docosahexaenoic acid; EPA, eicosapentaenoic acid; LA, linoleic acid; MUFA, monounsaturated fatty acids; SFA, saturated fatty acids; *n*-3 PUFA, sum of ALA, EPA, docosapentaenoic acid and DHA; *n*-6 PUFA, sum of LA, γ-linolenic acid, eicosadienoic acid, dihomo-γ-linolenic acid, ARA and docosatetraenoic acid. * Student’s* t*-test.

**Table 3:** Prevalence and risk of depressive symptoms according to tertile of intake and ratios of fatty acid types (adjusted hazard ratios (HR) and 95% confidence intervals).

	T1 (Low)	T2	T3 (High)	*p* for Trend *
SFA				
Median (range) of intake (g/day) †	11.1 (2.31–13.64)	16.0 (13.64–18.52)	21.8 (18.52–52.80)	
N with/without depressive symptoms ‡	173/604	167/611	175/605	
Crude HR (95% CI)	1.00 (reference)	0.89 (0.72–1.10)	0.91 (0.73–1.12)	0.35
Model I: Multivariate-adjusted HR (95% CI) §		0.93 (0.75–1.16)	1.00 (0.77–1.28)	0.97
Model II: Multivariate-adjusted HR (95% CI)		1.00 (0.79–1.25)	1.12 (0.86–1.46)	0.39
MUFA				
Median (range) of intake (g/day) †	14.2 (4.15–17.14)	20.0 (17.14–22.9)	27.2 (22.9–55.19)	
N with/without depressive symptoms ‡	168/609	174/604	173/607	
Crude HR (95% CI)	1.00 (reference)	0.94 (0.76–1.16)	0.91 (0.74–1.13)	0.38
Model I: Multivariate-adjusted HR (95% CI) §		1.04 (0.83–1.30)	1.08 (0.83–1.41)	0.58
Model II: Multivariate-adjusted HR (95% CI)		1.05 (0.83–1.33)	1.16 (0.88–1.53)	0.29
PUFA				
Median (range) of intake (g/day) †	9.6 (3.79–11.45)	13.0 (11.45–14.70)	16.9 (14.70–35.19)	
N with/without depressive symptoms ‡	166/611	178/600	171/609	
Crude HR (95% CI)	1.00 (reference)	1.00 (0.81–1.23)	0.91 (0.74–1.13)	0.38
Model I: Multivariate-adjusted HR (95% CI) §		1.08 (0.86–1.35)	1.04 (0.80–1.35)	0.78
Model II: Multivariate-adjusted HR (95% CI)		1.18 (0.94–1.47)	1.14 (0.88–1.48)	0.32
*n*-3 PUFA				
Median (range) of intake (g/day) †	1.6 (0.498–1.977)	2.3 (1.977–2.726)	3.3 (2.727–9.664)	
N with/without depressive symptoms ‡	186/591	177/601	152/628	
Crude HR (95% CI)	1.00 (reference)	0.92 (0.75–1.13)	0.74 (059–0.91)	0.005
Model I: Multivariate-adjusted HR (95% CI) §		0.94 (0.76–1.16)	0.75 (0.59–0.95)	0.018
Model II: Multivariate-adjusted HR (95% CI)		1.01 (0.82–1.25)	0.78 (0.61–0.99)	0.009
*n*-6 PUFA				
Median (range) of intake (g/day) †	7.7 (3.12–9.27)	10.5 (9.28–11.96)	13.8 (11.96–30.00)	
N with/without depressive symptoms ‡	171/606	165/613	179/601	
Crude HR (95% CI)	1.00 (reference)	0.89 (0.72–1.10)	0.93 (0.76–1.15)	0.52
Model I: Multivariate-adjusted HR (95% CI) §		0.96 (0.77–1.20)	1.08 (0.84–1.39)	0.54
Model II: Multivariate-adjusted HR (95% CI)		1.01 (0.81–1.27)	1.16 (0.90–1.49)	0.26
*n*-6:*n*-3				
Median (range) of ratios †	3.2 (0.805–3.961)	4.6 (3.962–5.258)	6.1 (5.262–16.849)	
N with/without depressive symptoms ‡	153/624	167/611	195/585	
Crude HR (95% CI)	1.00 (reference)	1.08 (0.86–1.34)	1.28 (1.03–1.58)	0.024
Model I: Multivariate-adjusted HR (95% CI) §		1.10 (0.88–1.37)	1.32 (1.07–1.64)	0.011
Model II: Multivariate-adjusted HR (95% CI)		1.11 (0.89–1.38)	1.36 (1.10–1.69)	0.005

CES-D, Center for Epidemiologic Studies Depression Scale; MUFA, monounsaturated fatty acids; N, number; SFA, saturated fatty acids; T, tertile; *n*-3 PUFA, sum of α-linolenic acid, eicosapentaenoic acid, docosapentaenoic acid, and docosahexaenoic acid; *n*-6 PUFA, sum of linoleic acid, γ-linolenic acid, eicosadienoic acid, dihomo-γ-linolenic acid, arachidonic acid, and docosatetraenoic acid. N, number; T, tertiles. * Based on Cox proportional hazards analysis according to tertile categories of fatty acid intakes. *p* for trend estimated by treating tertiles as ordinal variables for fatty acid intakes and ratios. † Values at baseline. ‡ New onset of depressive symptoms in follow-up period. A person with total CES-D score ≥16 is considered to have a significant depressive tendency. § Model I: Adjusted for sex, age, and energy intake. Model II: Adjusted for sex, age, energy intake, BMI, educational level, marital status, smoking status, alcohol consumption, energy intake, physical activity, employment status, CES-D score, and history of hypertension, hyperlipidemia, ischemic heart disease, stroke, and diabetes at baseline.

**Table 4:** Prevalence and risk of depressive symptoms according to the tertile of individual fatty acid intake (adjusted hazard ratios (HR) and 95% confidence intervals).

		T1 (Low)	T2	T3 (High)	*p* for Trend *
*n*-3 PUFA					
DHA	Median (range) of intake (mg/day) †	220.7 (4.2–344.4)	468.4 (344.6–627.3)	865.0 (627.4–3872.5)	
	N with/without depressive symptoms ‡	195/582	159/619	161/619	
	Crude HR (95% CI)	1.00 (reference)	0.80 (0.65–0.99)	0.79 (0.64–0.97)	0.023
	Model I: Multivariate-adjusted HR (95% CI) §		0.80 (0.65–0.99)	0.78 (0.63–0.97)	0.027
	Model II: Multivariate-adjusted HR (95% CI)		0.80 (0.65–0.99)	0.79 (0.63–0.98)	0.032
EPA	Median (range) of intake (mg/day) †	95.3 (2.1–164.8)	242.3 (165.1–341.8)	495.4 (341.9–2466.1)	
	N with/without depressive symptoms ‡	195/582	167/611	153/627	
	Crude HR (95% CI)	1.00 (reference)	0.87 (0.70–1.07)	0.77 (0.62–0.95)	0.016
	Model I: Multivariate-adjusted HR (95% CI) §		0.86 (0.70–1.06)	0.76 (0.61–0.95)	0.014
	Model II: Multivariate-adjusted HR (95% CI)		0.85 (0.69–1.04)	0.74 (0.60–0.93)	0.009
ALA	Median (range) of intake (mg/day) †	929 (289.1–1176.9)	1370.1 (1177.1–1604.8)	1919.4 (1604.9–4295.7)	
	N with/without depressive symptoms ‡	169/608	182/596	164/616	
	Crude HR (95% CI)	1.00 (reference)	1.03 (0.84–1.28)	0.90 (0.72–1.11)	0.31
	Model I: Multivariate-adjusted HR (95% CI) §		1.08 (0.87–1.34)	1.00 (0.78–1.27)	0.99
	Model II: Multivariate-adjusted HR (95% CI)		1.14 (0.92–1.41)	1.13 (0.88–1.44)	0.33
*n*-6 PUFA					
ARA	Median (range) of intake (mg/day) †	117 (13.1–144.6)	170.4 (144.6–197.3)	235.4 (197.4–612.5)	
	N with/without depressive symptoms ‡	163/614	188/590	164/616	
	Crude HR (95% CI)	1.00 (reference)	1.08 (0.88–1.33)	0.91 (0.73–1.12)	0.37
	Model I: Multivariate-adjusted HR (95% CI) §		1.14 (0.92–1.41)	0.98 (0.77–1.25)	0.87
	Model II: Multivariate-adjusted HR (95% CI)		1.17 (0.94–1.45)	1.04 (0.82–1.32)	0.75
LA	Median (range) of intake (g/day) †	7.5 (3.0–9.0)	10.2 (9.0–11.6)	13.6 (11.6–29.9)	
	N with/without depressive symptoms ‡	170/607	167/611	178/602	
	Crude HR (95% CI)	1.00 (reference)	0.92 (0.74–1.14)	0.94 (0.76–1.16)	0.54
	Model I: Multivariate-adjusted HR (95% CI) §		0.99 (0.79–1.24)	1.08 (0.84–1.39)	0.54
	Model II: Multivariate-adjusted HR (95% CI)		1.08 (0.86–1.35)	1.18 (0.92–1.52)	0.20

ARA, arachidonic acid; ALA, α-linolenic acid; CES-D, Center for Epidemiologic Studies Depression Scale; DHA, docosahexaenoic acid; EPA, eicosapentaenoic acid; LA, linoleic acid; N, number; T, tertile; * Based on Cox proportional hazards analysis according to tertile categories of fatty acid intakes. *p* for trend estimated by treating tertiles as ordinal variables for fatty acid intakes. † Values at baseline. ‡ New onset of depressive symptoms for follow-up period. A person with total CES-D score ≥16 is considered to have a significant depressive tendency. § Model I: Adjusted for sex, age, and energy intake. Model II: Adjusted for sex, age, energy intake, BMI, educational level, marital status, smoking status, alcohol consumption, energy intake, physical activity, employment status, CES-D score, and history of hypertension, hyperlipidemia, ischemic heart disease, stroke, and diabetes at baseline.
